# Supplementary material for: Evolution of conditional cooperation in public good games
Source: R Soc Open Sci. 2020 May 13;7(5):191567. doi: 10.1098/rsos.191567 (PMC7277267; doi:10.1098/rsos.191567)
Supplement: Supporting Information [file rsos191567supp1.zip › reroyalsocietyopensciencersos191567hasbeenunsubmit/Supporting.docx]

The function “ PGG_MAIN.m” is Matlab file implementation of the model.

The program file, “PGG_MAIN_RUN.m” is for creating data using PGG_MAIN function.

The program file, “randnlimt.m” is for creating mutations and used by PGG_MAIN.m.
